# Stakeholder Consultation Workshop on the Perceived Value of Thermostable Vaccines to Relieve Program Barriers: A Case Study from Côte d’Ivoire

**DOI:** 10.3390/vaccines12121414

**Published:** 2024-12-16

**Authors:** Anna-Lea Kahn, Dijana Spasenoska, Kouadio Daniel Ekra, Soplé Ruth Coulibaly, Kossia Yao, Sié Kabran Kouadio, Aminatou Sar, Joanie Robertson

**Affiliations:** 1 World Health Organization (WHO) Headquarters, Avenue Appia 20, 1211 Geneva, Switzerland; 2 Swiss Centre for International Health, Swiss Tropical and Public Health Institute, 4123 Basel, Switzerland; 3 Faculty of Medicine, University of Basel, 4056 Basel, Switzerland; 4 Department of Social Policy, London School of Economics and Political Science, London WC2A 2AE, UK; 5 Expanded Program on Immunization (EPI), Ministry of Health, Abidjan, Côte d’Ivoire; 6 World Health Organization (WHO) Country Office, Abidjan, Côte d’Ivoire; 7 United Nations Sustainable Development Group, Cotonou 01-3621, Benin; 8 PATH-Senegal, Dakar BP 15115, Senegal; 9 PATH-Headquarters, Seattle, WA 98121, USA; jrobertson@path.org

**Keywords:** stakeholder perceptions, thermostable vaccines, controlled temperature chain, innovation uptake, vaccine delivery, supply chain efficiencies, Côte d’Ivoire

## Abstract

Background: Persistent inequities in access to vaccinations pose challenges for immunization programs worldwide. Innovations facilitating vaccine delivery, such as leveraging vaccine thermostability through a Controlled Temperature Chain (CTC), have emerged as a potential solution to increase coverage in low- and middle-income countries (LMICs) countries such as Côte d’Ivoire, reducing dependence on the cold chain and improving vaccine delivery efficiency. However, the added value of thermostable vaccines and their integration into national immunization programs is under-recognized by stakeholders. This consultation aimed to convene key immunization stakeholders in Côte d’Ivoire in order to examine their perceptions regarding the value of vaccine thermostability to address barriers to outreach and equity in immunization programs. Methods: A novel workshop model involving structured group discussions was used to document the viewpoints of national stakeholders representing different areas of the immunization program. They prioritized barriers undermining coverage and equity in their country and explored the potential impact of CTC on the immunization program in the context of thermostable vaccines. The vaccines discussed were for Hepatitis B, Human Papillomavirus, and Meningitis. Results: The workshop outcomes highlighted the context and vaccine-specific variation of the importance of certain barriers, emphasizing the need for tailored strategies. The barriers considered most likely to be alleviated by vaccine thermostability were under the categories of human resource management, vaccine supply and logistics, and services delivery. The least relevant category of barriers concerned demand generation. Conclusions: The consultation provided valuable insights into stakeholder perspectives, priorities, and conditions for the effective integration of thermostable vaccines, informing future product development and policy decisions to optimize vaccine delivery and address immunization challenges in LMICs.

## 1. Introduction

Addressing persistent inequities in access to vaccinations, both between and within countries, continues to be one of the primary challenges of immunization programs around the world [1]. Delivering vaccines efficiently and effectively to all who need them continues to be an ambitious endeavor, associated with high costs and complexity, and undermined, in particular, by the need to keep vaccines in cool temperatures that require a well-managed and well-resourced cold chain. A notable solution are new vaccines with innovative attributes such as increased thermostability [2]. Thermostability means tolerance to heat exposure and lower dependence on a stringent cold chain. At the highest end of thermostability, vaccines would be taken out of the cold chain all together. Such innovation offers the potential for cost and time savings for vaccine delivery and better addressing the needs and realities of low- and middle-income countries (LMICs) [3,4]. 

Côte d’Ivoire is a country that experiences significant challenges maintaining the cold chain through the last mile of vaccine delivery, which can undermine access to more remote populations and compromise vaccination coverage [5]. These challenges are even more pronounced for vaccines that target persons outside of the traditional routine schedule during the first year of life, thereby often requiring community outreach strategies [6,7]. 

While several thermostable vaccines are already available, such as the vaccines against Meningitis A or Human Papillomavirus (HPV), their added value to the different components of national immunization systems is poorly documented, not broadly communicated, and insufficiently recognized by national immunization stakeholders [8]. This has contributed to the slow adoption of implementation practices such as the Controlled Temperature Chain (CTC), which take advantage of vaccine thermostability to permit limited storage and transportation outside of the standard cold chain [9]. The World Health Organization (WHO) defines the CTC as a vaccine management strategy allowing certain vaccines to be briefly kept at temperatures above the standard cold chain of +2 °C to +8 °C for a limited period under monitored and controlled conditions, as appropriate to the stability of the antigen (per confirmation through the WHO vaccine prequalification process). A CTC typically involves a single excursion of the vaccine into ambient temperatures not exceeding +40 °C and for a duration of a specific number of days, just prior to administration. The CTC is generally most effective in campaigns and special strategies where other vaccines that must be kept in the cold chain are not co-administered. In the face of low programmatic uptake and the implementation of CTC practices by immunization programs, vaccine manufacturers are not incentivized to meet CTC-qualification recommendations, undermining the availability and the potential of this innovation [10]. More guidance is required on when, where, how, and why vaccine thermostability and CTCs should be leveraged to facilitate vaccine deployment, such that uptake can increase, creating more demand and stimulating more compliance on the part of developers.

Having previously adopted a CTC approach during its introduction of the Meningitis A vaccination in 2014, Côte d’Ivoire already had direct experience and success with this approach. However, this constituted a time-limited strategy on account of this vaccine being then integrated into the routine immunization program for pediatric use after the initial introduction campaigns. With an evolving programmatic context and additional CTC-qualified vaccines becoming available or being in the pipeline, the question of whether the CTC is a well-suited innovation to Côte d’Ivoire’s needs and priorities re-emerged. National immunization programs are often faced with the possibility of adapting their systems to integrate such innovations, but it is difficult to know in advance whether the associated disruption and investment in time and energy will bring about the desired impact [11]. To evaluate the extent to which CTCs could improve the coverage and the equity of immunization programs, it was critical to engage relevant stakeholders to explore their views and perceptions on how CTCs might help overcome programmatic challenges. The WHO’s Innovation Framework offered a structured, transparent, and consensus-building approach to evaluating CTCs, which appealed to Côte d’Ivoire’s ministry of health, allowing it to both draw on previous experience and take into account current priorities and realities confronted by different regions of the country. 

This activity was established to examine the program realities underpinning decisions on the integration of CTC practices, when feasible, into national immunization programs. To determine both the optimal attributes of thermostable vaccines and the favorable conditions for their use through a CTC strategy, the perceptions, awareness, preferences, and priorities of stakeholders must be carefully considered. By discussing product attributes in the context of a country’s most pronounced immunization barriers, both the perceived relevance and the value of the innovation can be evaluated. This could also facilitate the identification of the key indicators against which to measure the extent of the impact of the innovative intervention. Discussing and documenting the perceived implementation challenges of national immunization programs in LMICs also offers important insights on unmet needs in terms of evidence, infrastructure, and guidance. Clarifying and documenting such perspectives and priorities can also inform the design and development of impactful products, policies, and strategies to best leverage vaccine thermostability. 

## 2. Approach and Methods

### 2.1. Selection of Workshop Participants

A workshop model relying on a qualitative, participatory approach was used. It involved group discussions with relevant national experts to examine how thermostable vaccines are valued in the context of the national immunization program. The seven thematic areas of the Expanded Programme on Immunization (EPI), as defined by the WHO (Table 1), served as the framework to ensure a comprehensive consideration of the full immunization system [12]. Relevant authorities within the national immunization program were asked to identify a balanced sample of participants from across key stakeholder categories, including a cross section of experts from regional or district levels offering perspectives from various geographic contexts, to take part in this workshop. Following detailed guidance on the broad and diverse set of professional profiles sought to be engaged, a total of 32 persons were nominated and agreed to participate, representing expertise across seven thematic areas of the EPI. The specific categories are listed in Table 1, along with an indication of their thematic areas of immediate relevance.

### 2.2. Methodology Used

**Figure 1:**
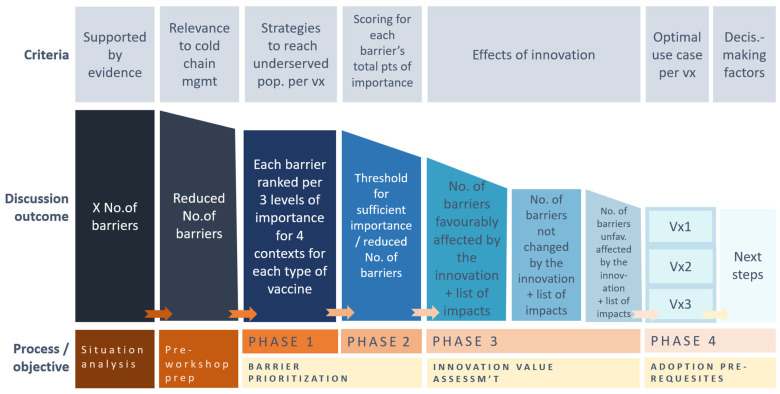
Criteria, subjects, and objectives across the four steps of the workshop.

A tailored discussion guide, using the Vaccine Innovation Framework derived from the WHO’s CAPACITI project [13,14], was used to structure a two-day workshop across four steps (Figure 1). The framework was adapted with context specific questions for Côte d’Ivoire and the vaccines of interest. The responses and discussion outcomes were recorded by a rapporteur into an Excel workbook and made available to the workshop participants during the course of the workshop and again afterwards for the purpose of validating all of the entries. A mix of plenary and break-out group discussions took place, depending on the step of the workshop and its respective objective. During the group discussions, efforts were made to ensure a balanced mix of stakeholders. 

The first step of the workshop, conducted in plenary, started with a presentation of Côte d’Ivoire’s identified immunization program barriers, grouped across each of the seven EPI thematic areas, relying on the evidence-based outputs of a previously conducted situation analysis. This list of barriers was then further pared down in quantity, based on their relevance to vaccine thermostability. The subsequently prioritized list was validated by the workshop participants and was the initial foundation for discussion.

Step 2 focused on the evaluation of the relevance and the importance of the identified barriers for different CTC-compatible vaccines and across different geographic settings. Three CTC-compatible vaccines were pre-selected by the Ministry of Health (MoH) of Côte d’Ivoire to be the focus for the workshop: the Human Papillomavirus Vaccine (HPV); the Hepatitis B (birth dose) vaccine (HepB-BD); and the Meningitis A (MenA) vaccine. To further prioritize the identified barriers for the vaccines discussed, the workshop participants were distributed across four groups: one per vaccine and a fourth considering the general EPI routine vaccines. Each group was assigned a previously prepared moderator and asked to select a rapporteur to record the group’s responses into a dedicated Excel worksheet. Their task was to define the underserved populations specific to the vaccine(s) their group was assigned to and the associated strategies to reach such populations. Each group then considered the list of barriers bearing these populations in mind and categorized each according to their relative importance across four geographic contexts: rural, urban, the Greater Abidjan area, and national-level considerations. The identification of the geographic contexts was made by the MoH staff during the workshop’s design and planning. 

The group moderators used four questions to assist the deliberations during the process of attributing relative importance to the following:

1. How sizeable is the impact of the barrier on coverage and equity?
2. Can the barrier be mitigated by changes in the vaccination program?
3. Are technological innovations likely to impact the barrier?
4. Are there other barriers of greater concern that have a greater impact on immunization coverage and equity?

The conclusions from the group discussions were validated during the plenary. Scores were assigned to each barrier in each setting, based on their importance level, where not important was scored 0, moderately important 0.5, and highly important 1. The final score of each barrier was the total sum across the settings. The barriers with the highest prioritization across settings were retained for the subsequent discussions in the workshop. Given that the maximum score attainable was 16, the threshold for high importance was set at 10–16. Ten was selected as the lower limit as it means the barrier had to be of high importance for at least 2 vaccines across 4 settings, and no lower than moderate importance for the rest. The thresholds served as guidance, which the group could accept or reject, with a consensus being sought for each prioritized status and diverging views recorded.

Step 3 of the workshop focused on the outcomes of vaccine thermostability and the innovative practice known as the Controlled Temperature Chain (CTC). Following a detailed presentation about the CTC as an innovation that can be applied to each of the three vaccines being examined, the workshop participants discussed each of the 15 prioritized barriers, aiming to determine the potential effect of the innovation on the barrier. The potential effects considered were the following: reduces/eliminates; increases; indirect advantage by increasing efficiency within the constraints of the barrier by reducing or eliminating other barriers; indirect challenge by amplifying or creating new barriers that create program inefficiencies that indirectly negatively impact the barrier; and no change. Each effect was then characterized in terms of favorable or unfavorable outcomes. Any additional qualifying comments, as considered necessary or of value, were also documented.

Following the discussion on the potential impact on program barriers, the workshop participants were divided into three groups and were invited to define the optimal use case for applying the CTC to each of the three vaccines. Each group provided feedback on all 3 vaccines. Among the categories of information requested were the following:

1. One or more potential use cases that could benefit from a CTC strategy, specific to how and/or when that vaccine is delivered;
2. Any relevant program barriers (even if not included on the prioritized list);
3. Enabling qualities of CTC in this context;
4. Required conditions for a CTC strategy to be adopted.

During the final step of the workshop, conducted in a plenary format, the participants were invited to comment further on the minimum conditions for adoption, as well as the likely criteria for any decision making on the CTC, including evidence requirements and policy and programmatic considerations. 

## 3. Results

The outcome of the situation analysis of the available evidence regarding the performance of the immunization program yielded 54 barriers to effective program implementation in Côte d’Ivoire (available on request from the national immunization program). At the start of the workshop, only 32 of these barriers were deemed pertinent to the storage, delivery, and handling of the three thermostable vaccines under discussion, validated as such by the workshop participants and, therefore, discussed during the workshop. It is worth noting that the prioritized barriers retained for subsequent discussions still cut across all seven thematic areas of the program, though 44% of the barriers were in the vaccine supply, quality and logistics, and service delivery categories. Only one relevant barrier was in the category of disease surveillance, and three were associated with demand generation.

The outcome of the context-specific prioritization exercise illustrated that the importance attributed to a given barrier can vary by vaccine and by context. Only one barrier, “Inadequate communication with community about immunization”, was considered of equally high importance for all the considered vaccines and across all the contexts. Four other barriers had a moderate importance across all the geographic contexts for the three CTC-relevant vaccines. In contrast, the same barriers were prioritized as being high importance for the EPI vaccines in general. As indicated in Table 2 below, barriers had a high importance most often for the rural context, when taking all four of the vaccine types into consideration. However, when disaggregated by the vaccine type, the barriers identified had a higher importance for Hepatitis B in the rural and urban settings. Among the three CTC-relevant vaccines, Hepatitis B (birth dose) vaccine delivery appears to be the most afflicted by the implementation barriers, irrespective of context. For the EPI vaccines, there was no difference in the importance of the barriers between rural and urban—the identified barriers were prioritized similarly. The stakeholders attributed the lower importance of the identified barriers to the vaccination coverage and equity in the context of the Greater Abidjan region.

The interpretation of the national context between the breakout groups varied, and to ensure consistency, we have excluded that prioritization from the future analysis.

Based on scoring thresholds, a total of 15 barriers were retained for the subsequent phase of discussions. These are listed in Table 3, grouped based on the respective EPI category with which the barrier is associated, demonstrating that the high-importance barriers to immunization are very diverse. One barrier, concerning budget reductions, was agreed by the workshop participants to lack relevance to thermostability considerations, despite scoring above the chosen importance threshold, and was, therefore, excluded from the third step of the workshop, based on the participating stakeholders’ request.

In step 3 of the assessment, the stakeholders agreed that 11 out of the 15 barriers could be positively affected by applying a CTC approach to vaccine delivery. Three barriers (appearing in the middle column in Table 3) were considered to not be impacted by the CTC in any way. Only inadequate communication with the community was assessed to be at risk of aggravation from the CTC, since it was believed that the lack of cold chain could be misinterpreted by some as an inadequate practice which undermines confidence in the vaccine. However, it should be noted that not a single barrier could be eliminated entirely thanks to the CTC, according to the workshop participants. 

Table 4 shows the favorable and unfavorable outcomes of CTC implementation, either directly or indirectly, on the barriers identified. It is particularly important to note that the implementation of the CTC resulting in easier transportation would positively impact five barriers, while improved efficiency for health workers would reduce three barriers. Less closed vial wastage and a lower risk of damaged vaccines due to the implementation of the CTC would address three barriers. However, the implementation of the CTC could result in some unfavorable immediate outcomes that would result in the worsening of certain barriers. For example, it might cause confusion for vaccine handling and, as a result, this would worsen the barrier related to the use of data for the planning of services as it might lead to incorrect reporting.

Considering the barriers discussed and the potential favorable and unfavorable outcomes of CTC implementation, the stakeholders discussed the potential use cases for CTC vaccines. Table 5 shows the various use cases and enabling factors for CTC use in those settings. For HPV, during mass campaigns and school-based strategies, CTC would enable better use of health workers’ time, easier transportation, and a higher number of girls vaccinated in one trip. Yet, successful implementation might be hindered by population mobility as it would be difficult to have an accurate microplan and to estimate the number of vaccines to take out of the cold chain. For the multivalent Meningitis vaccines still in the pipeline, the CTC would be particularly advantageous during large-scale introductions and catch-up vaccinations. Easier transport, leading to reductions in cost and time, would be an enabling factor for this use case. Finally, for HepB-BD, stakeholders identified health facilities without cold chains as the potential use case. The implementation of the CTC could be hindered by the lack of a cold chain for the longer storage of the vaccine and a lack of training. However, the flexibility in the vaccine distribution is seen as an enabling factor for the adoption of this use case. 

Limited insights were obtained about specific decision-making criteria and needs, with some emphasis placed on the importance of the National Immunization Technical Advisory Group (NITAG) to make a recommendation for the use of vaccines in the CTC, who would take into account the findings from this workshop. A clear interest was also expressed for additional evidence generated from implementation research to inform future programmatic decisions.

## 4. Discussion

Vaccine thermostability is an issue of supply chain logistics above all else, affecting how the vaccine is handled and managed through the supply chain and how well the vaccine quality can be maintained through mishandling or excursions from the cold chain. It is with this notion in mind that 54 confirmed immunization program barriers in Côte d’Ivoire were assessed for relevance to vaccine thermostability and the adoption of a CTC approach. A total of 22 of those barriers were considered not relevant to the CTC approach, as those were barriers relating to the program management and operations rather than the “last mile” delivery of the vaccines, where the CTC would be implemented, or because the barrier was not associated with any of the three vaccines being discussed. Of the remaining 32, most of them were distributed across three EPI categories: human resource management, vaccine supply, and quality and logistics, as well as service delivery. This suggests that it is particularly the countries that are confronted with problems in these categories that should consider CTCs as a potential source of relief.

The workshop discussions highlighted the importance of context when assessing immunization system barriers, noting variations per vaccine type and geographic setting, confirming that implementation strategies cannot be generalized. The identified barriers were consistently more pertinent to the rural context, regardless of the vaccine type. This is most likely because the most remote areas are the ones that prove the most challenging and require more resources to reach. In contrast, the identified barriers had a lower importance in the Greater Abidjan region, which can be attributed to the higher level of resources available in the capital city area, as well improved accessibility and transportation options, including public transport. Notably, the HepB-BD vaccine delivery faced the most significant implementation barriers across all of the contexts, which could be explained in the context of the complexity of timely vaccine delivery outside of a typical vaccination setting, be it in a maternity ward or in a home-birth setting.

The three vaccines on which this workshop focused target three very different types of populations. While HPV is delivered principally to adolescent girls (aged nine or above) in Côte d’Ivoire, through school-based delivery strategies, the HepB-BD vaccine is meant to be delivered to newborn infants within the first 24 h of life. The CTC-qualified MenA vaccine was delivered as part of an introduction program targeting individuals between the ages of 1 and 29 residing within the Meningitis belt. To reach these different populations, different strategies involving different actors are required, resulting in different unique indicators of success, defined by criteria varying from timeliness to cost-effectiveness or low wastage, to vaccination coverage. It is, therefore, not surprising that the importance of the barriers varies between the vaccines. This emphasizes the importance of tailored strategies for each vaccine that respond to the specific challenges encountered during their delivery. 

The narrow time-range within which HepB-BD is meant to be administered, coupled with the 20% rate of births occurring outside of a health facility [15], cause this vaccine to have a greater set of constraints in comparison to the other two vaccines under discussion. Similarly, in keeping with previously confirmed disparities in wealth between urban and rural households [1], the higher significance attributed to barriers for the less wealthy, rural context was expected.

Relying on the scoring thresholds and further deliberation triggering varied perspectives to be heard and considered, the workshop participants nevertheless reached a consensus around 15 barriers against which to evaluate a CTC approach. This outcome demonstrates how a transparent and multi-disciplinary discussion brings more credibility and confidence to the process by reaching a consensus despite highlighted differences.

The CTC was viewed as having a favorable impact on almost two thirds of the evaluated barriers, with these being expected to be reduced or indirectly influenced in a positive manner by CTC implementation. This suggests a strong case for its uptake in Côte d’Ivoire. However, it is important to note that none of the barriers could be entirely eliminated by the CTC, according to the workshop participants, indicating that while the CTC may offer a viable solution to bring collective relief to a constrained program, it is not a single solution to any one problem. Furthermore, the potential gains from the CTC are not without their trade-offs, which include the need for training, additional monitoring, and supervision to avoid potential confusion in vaccine handling and the requirement for the procurement of temperature monitoring tools. To fully experience the favorable impact of the CTC on the identified barriers, it is essential to address these concerns and provide appropriate guidance to mitigate the potential disadvantages of CTC implementation.

Through the definition of the use cases, the workshop participants were able to easily envision a role for the CTC in the delivery of each of the three vaccines discussed. Particular enthusiasm was noted for the HPV vaccine, most likely as this is the only vaccine with a readily available CTC-qualified product. It is also the one which the country not only has ample implementation experience with but also has recently elevated to a priority status for improving its coverage rates. These discussions shed light on the enabling qualities of the CTC and the required conditions for its adoption. The opportunity for participating decision makers to directly hear the perspectives of different experts from across Côte d’Ivoire’s immunization system on how priority issues can be specifically addressed by the CTC also made this workshop uniquely compelling and informative, creating a rare forum for dialogue on and insight into this topic that may facilitate future policy considerations and the eventual uptake and expansion of CTC strategies in the country. This also speaks to the value of this approach for considering and assessing innovative interventions, both for Côte d’Ivoire and other LMICs. 

The stakeholder consultation provided valuable insights into the barriers, priorities, and the potential impacts of CTC implementation in the immunization program in Côte d’Ivoire. The findings contribute to the understanding of the needs, preferences, and priorities of the stakeholders regarding the integration of thermostable vaccines into the national immunization system, information that has value at the national level, as well as the regional and global levels, to inform research agendas and product development. Insights into the barriers with the greatest potential for beneficial impacts from the CTC also enable the designation of more suitable metrics by which to measure the success of the CTC, which cannot and should not be reduced just to increases in the vaccination coverage. The prioritized barriers and the assessment of the potential impacts of the CTC provide a foundation for designing evidence-based interventions and strategies to enhance vaccine delivery, particularly in underserved populations and challenging geographic contexts. These results are also expected to inform upcoming decision making in Côte d’Ivoire, be it to define specific evidence needs, designate impact benchmarks, or develop policy. They can guide the development of strategies to improve vaccine delivery and overcome implementation challenges for the three discussed vaccines. By addressing these challenges and leveraging the potential of thermostable vaccines, Côte d’Ivoire can enhance its immunization program’s effectiveness and reach, particularly for hard-to-reach populations and those in remote areas.

## 5. Limitations

This analysis reflects the perceptions and opinions of key stakeholders in Côte d’Ivoire, for a specific context. Thus, these views can change and evolve over time. However, there are two measures the authors have taken to address this potential limitation. First, the list of barriers, which underpins the discussion, was derived from existing documented evidence in the country, and it is not based on the opinions of the experts. Second, a detailed Excel-based guide was used to lead and document the discussion, allowing for the reproducibility of the discussion in other settings or in different points of time. Thus, although the findings reflect perspectives, the transparent and systematic documentation of the discussion allows for its comparability with findings from other consultations following the same methodology across countries, across time and across stakeholders.

## 6. Conclusions

Overall, the stakeholder consultation highlighted the value of implementing the CTC in Côte d’Ivoire’s immunization program, signaling to decision makers that this innovative approach to vaccine delivery is worth considering for each of the three types of vaccines discussed and highlighting the specific contexts where it could offer the most benefit. The prioritized barriers and their association with specific thematic areas indicate the complex challenges faced by the program. The CTC was perceived as a favorable approach that can address many of these barriers and offer several advantages, such as improved efficiency, reduced costs, and increased vaccine availability.

The identified use cases for specific vaccines demonstrated the applicability of the CTC in diverse scenarios, enabling immunization in hard-to-reach populations and addressing cold chain limitations in various settings. These use cases, along with the enabling factors, provide valuable insights for policymakers and program managers, paving the way for a more informed consideration of the legitimate trade-offs associated with CTC implementation. This should eventually ensure optimized impacts when planning, implementing, and documenting CTC interventions tailored to specific vaccine types and contexts.

## Figures and Tables

**Table 1:** Immunization program stakeholders and their respective relevance to the seven thematic areas of an Expanded Programme on Immunization (EPI).

		The 7 Thematic Areas of an Expanded Programme on Immunization (EPI)						
EPI Stakeholder categories		**Program Management and Financing**	**Human** **Resource Management**	**Vaccine** **Supply,** **Quality, and** **Logistics**	**Service** **Delivery**	**Immunization Coverage and Adverse Events Monitoring**	**Disease** **Surveillance**	**Demand** **Generation**
	Immunization program leadership	✓	✓	✓	✓	✓	✓	✓
	NITAG * members	✓				✓		
	logisticians			✓	✓		✓	
	HCWs */ managers		✓	✓	✓	✓	✓	✓
	Researchers/ academia				✓	✓	✓	✓
	WHO	✓	✓	✓	✓	✓	✓	✓
	UNICEF	✓		✓	✓			✓
	Relevant NGOs *	✓	✓	✓	✓	✓	✓	✓
	CSOs *	✓			✓	✓	✓	✓

* NITAG: National Immunization Technical Advisory Group; HCW: Health Care Worker; NGO: Non-governmental Organization; CSO: Civil Society Organization.

**Table 2:** Rate of barriers classified as high importance.

	URBAN	RURAL	GR. ABIDJAN
EPI Vaccines	75% (N = 24/32)	75% (N = 24/32)	59% (N = 19/32)
HPV vaccine	28% (N = 9/32)	25% (N = 8/32)	22% (N = 7/32)
Men-A vaccine	19% (N = 6/32)	22% (N = 7/32)	19% (N = 6/32)
Hep-B BD vaccine	56% (N = 18/32)	75% (N = 24/32)	53% (N = 17/32)

**Table 3:** How barriers prioritized in step 3 are expected to be impacted by the CTC across the EPI categories.

EPI Category	Prioritized Barriers		
	Likely to Be Positively Affected by CTC	Considered not Impacted by CTC	Potentially Worsened by CTC
Program Management and Financing	Updated strategic and operational plans and strategies for hard-to-reach areas or children who missed their RI doses	Adequate national immunization laws and policies regarding immunization practices	
	Insufficient budget for immunizations at the sub-national level	Slow or inadequate provision and disbursement of funds from the central level to sub-national and local needs	
HR Management	Poor staff motivation		
	Suboptimal number and quality of supervisory visits		
	Inadequate training to prepare health staff in immunization or new vaccines		
Vaccine supply, quality, and logistics	Inadequate quantity of functional cold chain equipment		
	Weak transport system (lack of vehicles/fuel/maintenance)		
	Vaccine wastage due to heat or freezing exposure, inappropriate storage conditions		
Service Delivery	Long distances and travel time lead to poor access to health facilities		
	Poor integration of EPI strategies with other primary health care (PHC) services		
Coverage and AEFI monitoring	Private (profit/not for profit) providers pose challenges to immunization delivery or interpretation	Data on service utilization and equity are not optimally used for service delivery planning	
Demand generation			Inadequate communication with community about immunization

**Table 4:** Distribution of favorable and unfavorable outcomes of CTC implementation and respective number of barriers affected. Note: more than one outcome could be selected per barrier.

Type of Outcome	Outcome	No. of Barriers Affected
Favorable Outcomes of CTC Implementation	a. Easier transportation	5
	b. Improved use of time for health workers (improved efficiency)	3
	c. Reduced burden on health workers (less constraints associated with ice pack preparation, transport, tracking; more flexibility in the field)	3
	d. Lower infrastructure and delivery costs	3
	e. Reduced closed vial wastage	3
	f. Lower risk of damaged vaccines	3
Unfavorable Outcomes of CTC Implementation	a. Need for training	5
	b. Requires additional monitoring and supervision	4
	c. Might cause confusion for vaccine handling as other non-thermostable vaccines might be taken out of the cold chain	3
	d. Extra procurement for temperature monitoring tools	1

**Table 5:** Use case for CTC by vaccine.

Vaccine	Use Case For CTC	Factors Hindering the Use Case	Factors Enabling the Use Case
HPV	Mass campaigns, school-based strategies, community-based strategies (finding/catching up out-of-school girls)	Displaced or in-transit populations Girls not in school Missed opportunities Teacher reluctance	Better use of health workers’ time Easier transportation More people vaccinated in one trip
Men A-C-W-Y (X) *	During large-scale introduction campaigns or catch-up vaccinations	Displaced or in-transit populations Difficult access areas Missed opportunities Insufficient number of refrigerators/cold chain equipment in working order	Better use of health workers’ time Less closed vial wastage Easier transportation Reduced cost of transport More people vaccinated in one trip
Hep-B Birth dose	Maternity wards without cold chains, private facilities without cold chains, home-births	Absent cold chains Lack of training for private (and public) agents Difficult access areas	Flexibility in vaccine distribution timing

* For the purposes of forward-looking discussions on the use cases, the multivalent vaccine was used rather than Meningitis A, which previously served as the basis of workshop discussions about vaccination experiences to date.

## Data Availability

All the data supporting the conclusions of this case study are provided, though a more detailed record of the workshop discussion outcomes is available upon request to the corresponding author.
